# So what? Elevating the impact of implementation science

**DOI:** 10.1186/s43058-025-00831-9

**Published:** 2025-11-27

**Authors:** Ross C. Brownson, Juliet Iwelunmor, Thomas A. Odeny, Enola K. Proctor, Elvin H. Geng

**Affiliations:** 1 Prevention Research Center, School of Public Health, Washington University in St. Louis, One Brookings Drive, Campus Box 1196, St. Louis, MO 63130 USA; 2 Alvin J. Siteman Cancer Center, Washington University School of Medicine, Washington University in St. Louis, St. Louis, MO 63130 USA; 3 Division of Infectious Diseases, Department of Medicine, Washington University School of Medicine, Washington University in St. Louis, St. Louis, MO 63110 USA; 4 Division of Oncology, Department of Medicine, Washington University School of Medicine, Washington University in St. Louis, St. Louis, MO 63110 USA; 5 Brown School at Washington University in St. Louis, One Brookings Drive, Campus Box 1196, St. Louis, MO 63130 USA; 6 Division of Infectious Diseases, Department of Medicine and Center for Dissemination and Implementation, Washington University School of Medicine, Washington University in St. Louis, St. Louis, MO 63110 USA

**Keywords:** Clinical practice, Equity, Implementation science, Population health, Scale-up

## Abstract

**Background:**

Given the substantial public funding of health-related research, tangible benefits of this support must be demonstrated. Implementation science provides actionable methods to enhance population health, reduce health inequities, and guide effective public health and clinical practices and policies. We must elevate the notion of impact (the “so-what gap”) and the role of implementation science, particularly in university settings.

**Main text:**

We distinguish between scientific output and impacts. Impacts in implementation science are commonly defined as improvements in health outcomes, quality of life, quality of services, or policy change. In contrast, traditional academic outputs, such as citation counts and grant awards, hold minimal, direct societal relevance. Principles of audience segmentation (partitioning the target audience for dissemination and implementation into smaller groups by meaningful distinctions), which are increasingly applied in implementation science, can enhance impact. We highlight trade-offs in enhancing the focus on impact across multiple categories (e.g., accountability, evaluation). We describe four essential domains of implementation impact: speed of research translation, sustainability, de-implementation, and equity. Multiple examples, across diverse topics, illustrate these domains (e.g., HIV treatment, use of community health workers). To boost impact via more active dissemination and implementation of research findings, we provide ideas within five categories: (1) co-production of knowledge, (2) tailored dissemination, (3) organizational support, (4) capacity building, and (5) implementation metrics.

**Conclusions:**

Generating new research knowledge does not guarantee societal impact. For implementation science to become more relevant to societal needs, enhancing and evaluating its impacts matter; otherwise, systemic changes required in institutions will continue to evolve slowly. We argue that impactful implementation science involves developing new skill sets and uncovering meaningful work that changes the field while adopting a collaborative working approach with individual researchers, their organizations, funders, and the communities they aim to benefit. Navigating the hurdles and translating research into practice and policy can amplify societal impact, making implementation science more applicable, accessible, and equitable for all.

Contributions to the literature

- Impact has different meanings for different audiences, and university-based researchers mainly focus on academic outputs (e.g., journal impact factors, grants) that represent knowledge generation itself and may have attenuated relevance to the rest of society.
- Actions to enhance implementation science impact should consider the trade-offs in shifting the research enterprise.
- Impactful implementation science emphasizes four essential implementation domains (speed of research translation, sustainment, de-implementation, and equity).
- Implementation science impacts can be improved by both incremental and transformational change.

“What you do makes a difference, and you have to decide what kind of difference you want to make.”

– Jane Goodall

## Introduction

The substantial public investment in health-related research must ultimately yield tangible benefits to population health and society [1]. Globally, funders often place a high priority on research; they also increasingly want more information on the effectiveness of approaches and societal benefits, including tangible impacts on population health [2–6]. In a survey of 31 health funders across the globe, van der Linden and colleagues found a high priority for dissemination and implementation (D&I) of research to increase value and impact [4]. The team also identified the challenges in identifying and applying successful approaches for D&I, along with metrics for assessing impact [4].

Much clinical and public health research yields tangible benefits for practice, policy, and society. For example, one of the significant achievements of public health and medical science is reducing the burden of vaccine-preventable diseases (e.g., polio eradication; control of cervical and liver cancer) [7, 8]. Despite such evidence, the gaps that remain between what could work to improve health and what is applied in public health practice, clinical practice, and policy change are the focus of growing attention and dissatisfaction [9–11]. The substantial benefits of bridging this chasm remain unrealized and are higher in many parts of the world. For example, scaling-up just 10 community-level interventions in five East African countries (Burundi, Kenya, Rwanda, Uganda, and the United Republic of Tanzania) could avert approximately 75,000 child deaths [12].

The science of implementation focuses on increasing the adoption, implementation, and sustainment of EBPPs, ensuring that implementation strategies align with the context of the setting or population, and improving equity across settings. To date, implementation science literature has focused primarily on “what” and “how”—defining the field’s key concepts and determining questions about how strategies work. In brief, “knowing what to do” does not ensure “doing what we know” [13]. This so-called “know-do gap” is partially due to ineffective implementation and sustainment of evidence-based programs and policies (EBPPs) [14]. Now is the time for implementation science and the related research community to ask questions of “so what?”—emphasizing and crafting paths to impact that demonstrate a more tangible return on investment.

Therefore, in this article, we describe (1) implementation science and potential impacts; (2) definitions of impact, trade-offs, and audience differences; (3) the distinctive role of selected implementation science domains to enhance impact; and (4) recommendations for achieving greater and more equitable impacts.

## Implementation science and its impacts

Implementation science focuses on advancing impact, which is crucial for transforming scientific discoveries into actionable strategies that improve population health, reduce health inequities, and inform effective public health, clinical practice, and policy [15, 16]. The growth of implementation science has been a critical shift in biomedical sciences [17]. The focus on the impact of implementation science coincides with a movement toward consequentialism [17], aligning with the utilitarian ethic of maximizing positive impacts and relying in part on implementation science [18]. The shorter-term impacts of implementation science may include intentions to use evidence in program or policy development, as well as increased self-efficacy among practitioners when using or finding evidence. In the medium term, indicators of impact may include greater use of EBPPs in public health settings or improvements in clinical practice [19]. Long-term impacts include well-known outcomes such as disease burden (e.g., years of life lost) or economic value (e.g., cost-effectiveness) [20].

A focus on policy implementation is crucial because policy has a profound impact on population health [21]. However, policy implementation is understudied [22], and the policy process has distinctive implementation properties (less control over timing, the need for local data, the role of ideology, and special interests) [23]. The scale-up of the US President’s Emergency Plan for AIDS Relief (PEPFAR) is an example of a significant advance in addressing the global HIV/AIDS epidemic. In PEPFAR, high incidence and mortality from HIV/AIDS (the problem), funding for life-saving antiretroviral therapy (the policy), and effective advocacy coupled with bipartisan support (the politics) converged [24].

## Defining impacts, trade-offs, and audiences

Many scholars and organizations call for a broader, more real-world view of research impact [25, 26]. A standard definition of impact is “a significant or major effect” [27], which has a general meaning across all of society. Selected large-scale efforts and reviews to better document research impact outside of academe have developed definitions [28–33]. The most common elements across various definitions include quality of services, policy change, health outcomes, quality of life, economic benefits, environmental changes, and social/cultural impacts [32, 33]. Other elements include avenues to change (attitudes, awareness, funding) across multiple levels (local, regional, national, and international) [33]. While not explicit in most definitions, impact can be positive or negative. For example, community development to enhance the built environment can result in benefits (e.g., greater walkability, economic growth) and unintended consequences (e.g., gentrification, displacement of long-time residents) [34]. In Africa, traditional indoor cooking with biomass fuels produces smoke, which, though detrimental to respiratory health, has an observed repellent effect on mosquitoes [35]. Consequently, the adoption of smokeless cooking devices, while significantly reducing indoor air pollution and associated respiratory ailments, could potentially increase exposure to mosquito-borne diseases.

### Trade-offs in increasing a focus on impact

Increasing the focus on impact in implementation science involves some changes in thinking and practices. Every decision in implementation science involves one or more trade-offs (comparative gains and losses). While not covering these topics in depth, we offer a series of trade-offs that are often driven by available resources, organizational priorities, skill sets, policy, and politics (Table 1). External accountability pressures researchers to demonstrate medium- to long-term societal impact, sometimes in conflict with shorter-term academic incentives. Definitions of impact vary across disciplines and community segments. Projects should focus on long-term societal outcomes, but these outcomes are often beyond the scope of research timelines. Equity-focused, high-impact strategies may neglect marginalized groups needing new resources and tailored approaches. Enhanced co-production and storytelling may improve impact documentation but require skills beyond traditional research competencies, risking increased bias and burden on academic freedom. In practice, these trade-offs are seldom either/or considerations; rather, as researchers strive to enhance impact outside of academic settings, they should keep these and other trade-offs in mind and seek to maximize the return on investment for their scholarship.

**Table 1:** Trade-offs of increasing the focus on impact in implementation science

*Category*	*Pro*	*Con*
*External Accountability*		
Societal impact	Research should more fully document societal impacts Stakeholders (funders, partners, residents) expect a return on investment from research	A focus on societal impacts does not comport with academic incentives for promotion and tenure; research may be subject to political review as social forces act through politics
Social contract	Publicly- and privately funded researchers have a social contract and an obligation to show societal benefits	Every academic discipline has a different definition of impact, making it challenging to fulfill a social contract Impact is defined differently across community segments with distinct social and political values
*Focus and Scope*		
Project focus	Impact beyond standard academic metrics (grants, publications) should be a focus of every research project	The time horizon for societal impacts is long, much longer than the duration of a typical research project
Equity focus	More attention is needed on high-impact, scalable strategies in implementation science to address inequities Lessons about equitable implementation are available across countries, settings, and populations	High-impact approaches may leave out marginalized groups who need more tailored and resource-intensive strategies Groups marginal to science may not hold social power and privileged public discourse (e.g., news media, social media)
*Engagement and Communication*		
Co-production	Impact is enhanced by co-producing implementation science with practitioners, policymakers, and/or payers	Participatory research is time-intensive and beyond the scope of many research projects
Storytelling	Stories backed up by data (and data backed up by stories) can better document the impact of implementation science	Researchers are not well-qualified to tell stories Storytelling requires audience segmentation and knowledge, which is a profession in and of itself (e.g., journalism)
*Evaluation and Reporting*		
Standardization	Standardized frameworks help to show the value of implementation science	Standardized approaches in measuring impact will stifle innovation and impede academic freedom; increase the burden of science
Measurement	There are multiple methods for measuring the societal impacts of implementation science	There is a high potential for bias in the assessment of impact, in part because we measure the elements that are easy to measure (publications)

It is also important to consider impact trade-offs in light of the social context for research. Socially responsive research is essential, yet it also carries risks [36]. Social forces—particularly when amplified through political mechanisms—can shape science in ways that undermine its integrity. In the current climate, scientific discourse is subject to anti-vaccination ideologies or partisan agendas at the highest levels of government. If science is expected to be accountable to society, and government is presumed to represent society, then the rise of “cancel science” movements illustrates how social influence can produce instability and whiplash in the scientific community.

### Audiences and audience segmentation

Impact has different meanings for different audiences. University-based researchers often prioritize academic outputs (e.g., citation rates, journal impact factors, grants) that represent knowledge generation itself, which may have an attenuated direct relevance to the rest of society. As appropriate for specific areas of scholarship, researchers often prioritize discovering new knowledge (rather than applying it). Several factors drive the focus on academic metrics among researchers: academic incentives (promotion/tenure guidelines), ease in measuring outputs (grants, publications), difficulty showing the causal attribution of research impacts to practice and policy change, and lack of skills among researchers in non-academic dissemination. It is also essential to focus on the impact of scholarship rather than only on published research. White papers or testimony to policy bodies may be highly influential even if the relevant scholarship is not published in journal articles [37].

Audience segmentation helps enhance impact. Audience segmentation, which originated in business and social sciences, involves partitioning the target audience for D&I into smaller groups by meaningful distinctions (e.g., demographic, psychographic) [38–41]. Segmentation helps to ensure that research products meet the unique requirements of market segments, in part by delivering tailored messages to enhance the timeliness, relevance, and usefulness of research findings. Audience characteristics (e.g., motivations, social influences, time urgency) are considered to fine-tune messages and dissemination strategies [42]. Table 2 summarizes key audience differences regarding the impact of research. Practitioners and policymakers value practical ways to apply knowledge to their settings, often with adaptation for local relevance [43–45]. Given the many audiences for research uptake, we do not suggest that researchers should alone fill the dissemination void. Researchers can and should often partner with audiences in Table 2 to form a bridge between the generators of knowledge and those most likely to affect change and impact.

**Table 2:** Audience segmentation for more impactful implementation science

*Segment/actors*	*Relevant characteristics*	*Messages and impacts*	*Channels*^*a*^
Health researchers	• Deep knowledge of specialized issues • High commitment to health • Long horizons in the research process • Main incentives to publish research, obtain grant funding • Few incentives to disseminate research outside the scientific world	• Make a difference in society • Improve health equity • Enhance resources for research • Evidence being used to guide practice • Enhanced capacity among research partners	• Journal articles, especially in leading journals • Professional associations/meetings • Leading scholars (opinion leaders) • Study participant feedback
Public health practitioners	• High commitment to health • Wide range of professional backgrounds • Access to summaries of evidence but often not the original research • Mid to long-term horizon for impacts	• Make a difference in society • Improve health equity/social justice • Prevention saves lives • Enhance resources	• Leadership meetings, preferably face-to-face • National government agencies • Professional associations • Brief summaries of evidence • Social media
Clinical practitioners	• High commitment to health • Narrow range of professional backgrounds • Time urgency • Short-term horizon for impacts	• Improve patient care • Improve quality and safety of care • Improve health and well-being • Improve health equity • Enhance efficiency of care	• Journal articles • Professional associations • Professional conferences • Brief summaries of evidence • Social media • In-service training
Policymakers (Big P policy, elected officials)	• Variable commitment to health (often limited knowledge across many issues) • Wide range of professional backgrounds • Influence of political parties, ideology • Some rely heavily on staff • Trust and credibility built on personal relationships • Short-term horizon for impacts	• Serve constituents • Improve peoples’ lives • Create return on investment • Pressure of re-election	• Real-world stories, • Brief summaries of evidence • Delivery of messages by opinion leaders
Staff members for elected officials	• Variable commitment to health (often limited knowledge across many issues) • Wide range of professional backgrounds • Will read longer reports • Trust and credibility built on personal relationships • Short-term horizon for impacts	• Serve elected official(s) • Serve party priorities • Create return on investment	• Longer summaries of evidence • Experiences from similar jurisdictions • Delivery of messages by opinion leaders
Agency and system administrators, directors, and leaders (small p policymakers)	• High commitment to health (if in a health agency/ system) • Narrow range of professional backgrounds • Time urgency • Short to medium horizon for impacts • Among those in government, there are limits on advocacy	• Improve care/population health • Seek congruence of outcomes with strategic plans/agency aims • Manage budgets effectively and efficiently	• Longer summaries of evidence for selected issues • Delivery of messages by opinion (other system) leaders • Professional associations • Feedback from embedded researchers
Community members and community partners	• Variable commitment to health • Value different types of ‘knowledge’ and ‘evidence’ regarding health • Impacted by personal or familial experiences as patients • Wide range of professional backgrounds • Short-term horizon for impacts	• Provide tangible benefits or relevance to self, family, community • Value as a service or resource to the community • Seek not to incur high costs or financial burden	• Local media channels (including social media) • Real-world stories • Culturally appropriate media (local papers, radio) • Local community leaders (opinion leaders) • Economic impacts of interventions
Research payers	• High commitment to public health • Priority on evidence • Impacted by advocacy groups, advisory boards • Typically, former researchers or policymakers • Intermediate and long-range horizons	• Return on investment of research dollars • Balance a portfolio of research types • Accountability to elected officials, ministers of health, boards • Seek congruence of outcomes with strategic plans/agency aims • Manage budgets effectively and efficiently	• Researchers’ annual and final reports • Peer-reviewed publications • Brief summaries of key findings

^a^The route of message delivery to reach the audience segment

Adapted and expanded from Brownson et al. [43], Shato et al. [44], and Shelton and Brownson [45]

The impact of translational research on policy and practice can be defined in ways beyond individual-level health outcomes. Nearly 50 years ago, Weiss encouraged using more flexible and exact concepts about the policy environment [46]. She argued that research can change policy and practice by prompting a new conceptualization of a problem in a policy community. For example, Farmer’s work in Haiti used a single-arm case series of HIV treatment success without sophisticated laboratory support [47]. These efforts helped the global public health community reimagine HIV treatment as a public health challenge rather than a medical care issue. In some ways, Weiss's model requires a paradigm shift in research known for its impact. Research on HIV treatment changed the perception that HIV had to be treated with highly specialized doctors and sophisticated monitoring. While no one has adopted the original Farmer model at a larger scale (e.g., no laboratory monitoring), the research has changed mindsets and led to global investments that would have been impossible.

## Implementation science domains to enhance impact

When research addresses real-world challenges, is conducted with rigorous methods, and is disseminated to key leaders and partners, beneficial impacts occur [48, 49]. Implementation science methods underscore the impact of research with efforts to close the so-what gap [50].

While not an exhaustive list, to illustrate documented and potential impacts of implementation science, we describe four exemplar implementation topics (speed of research translation, scale-up and sustainment, de-implementation, and equity) along with brief examples (Table 3) [51–55, 57, 58]. We chose these topics because population health impact is maximized when EBPPs are implemented with speed [59], sustained over time [62], with unnecessary or harmful practices de-implemented [63], and equity prioritized to ensure all populations benefit [64]. These four domains are also priority action areas for the broad field of implementation science [59–61]. Each example illustrates how implementation science concepts and methods contributed to impact, including core principles, the context for implementation, and how approaches were tailored to various audiences.

**Table 3:** Selected implementation science topics^a^

*Topics*	*Definition*	*Examples of impact*
Speed	Time of research translation is measured in multiple ways: time in a translational stage; time to achieve an implementation milestone; and time to achieve a predefined outcome (service system, health outcome)	Collaborative care for depression: The DIAMOND Initiative (Depression Improvement Across Minnesota–Offering a New Direction) was implemented in 75 primary care clinics in two years [51] COVID-19 vaccines: The rapid development and distribution of COVID-19 vaccines significantly mitigated the impact of the pandemic
Sustainability	Sustainability is the extent to which an evidence-based practice can deliver benefits over an extended time	School nutrition in Australia: A multi-strategy intervention has been scaled to over 2,000 schools and sustained for nearly 20 years [52] Tobacco control in California: A 30-year experience in scaling up tobacco control policies has shown a return on investment of 231 to 1 in direct medical expenditures [53]
De-implementation	The process of stopping practices that are ineffective or harmful, not the most effective or efficient (including cost-effective), or no longer necessary; maintaining ineffective practices wastes precious resources	Chest X-rays to screen for tuberculosis: Routine X-ray screening in low-risk populations has been discontinued due to low effectiveness and potential for harm in low-risk populations [54] Hormone replacement therapy (HRT) for postmenopausal women: HRT was widely prescribed for the prevention of heart disease and osteoporosis, but large studies subsequently showed more risks than benefits [55]
Equity	Everyone has a fair and just opportunity to be as healthy as possible [56]. Equity in implementation involves social justice and requires greater attention to the needs, cultures, and histories of communities being served	Housing programs for low-income families: Initiatives to provide safe and affordable housing have improved health outcomes (respiratory health, mental health) [57] School meal programs in the US: School meal programs improve nutritional intake and academic performance among children from low-income families [58]

^a^This is not a comprehensive list of topics, but rather examples where progress has been made to varying degrees and are priorities for implementation science [59–61]

### Speed of research translation

The speed at which actionable evidence is implemented has sizable implications for impact [59]. Over two decades ago, it was estimated that EBPPs take an average of 17 years to affect practice across multiple diseases and risk factors [65, 66]. More recently, Khan and colleagues studied five EBPPs in cancer control: mammography, clinicians’ advice to quit smoking, colorectal cancer screening, HPV co-testing, and HPV vaccination [67]. They found that the time from publication to implementation ranged from 13 years (advice to quit smoking) to 21 years (mammography), averaging 15 years. The challenge is conceptually simple: getting what works to the people who need it with the greatest speed and efficiency [68]. The framework to assess the speed of translation is a comprehensive model for describing and addressing the determinants of the pace of implementation [59]. We acknowledge that in some cases, speed may not be warranted (e.g., the evidence base is still developing, the safety of a medical treatment has not been thoroughly tested) [59].

#### Example: HIV treatment

*Context*: Historically, it was believed that starting HIV antiretrovirals should be a cautious and sometimes slow process, often involving weeks or months of counseling to prepare patients. However, emerging evidence in the early 2010s demonstrated that rapid initiation of treatment had clinical benefits previously unrecognized by the scientific community (e.g., fewer AIDS-related events even among patients not exhibiting symptoms at the time of assessment, reduced risk of HIV transmission) [69]. Implementation science illustrated whether rapid initiation was possible and how to do so. Rapid antiretroviral therapy (ART) initiation required re-thinking assumptions and standard practices such as multiple adherence counseling sessions (the norm in Africa) and the perceived need for pre-treatment laboratory studies (the norm in the US). Several studies published in 2015 and 2016 showed not only outcomes with rapid initiation of treatment, but also demonstrated changes in workflows, clinical assessments, training, and incentives in the health system that enabled rapid ART initiation [70–72].

*Impact*: These studies, in part, led the World Health Organization to make a “rapid policy recommendation” on July 1, 2017 [73]. These policies were developed through a guideline review committee and input from opinion leaders, program leaders, and scientists worldwide. In many regions where HIV prevalence is high, this policy was adopted quickly and comprehensively, and by 2018, the vast majority of patients in high-prevalence settings in Africa, as well as most settings in the United States, were starting HIV medication on the day of or within a few days of diagnosis [74].

*Key lessons*: The shift toward rapid HIV treatment initiation challenged prior, more cautious approaches. While science often moves at a deliberate pace, this case study shows that shifting norms about treatment, the urgency of a health issue, and the opening of a policy window [23] can quickly impact clinical treatment protocols. Policy change is often most effective when a diverse group of stakeholders is involved in the process.

### Sustainability

Sustaining interventions ensures that they reach a larger portion of the population over time. This is essential for achieving widespread equity with public health impacts. Sustainability refers to the lasting use of an EBPP whose benefits persist over an extended time, often after external support from a funder ends. Scheirer and Dearing suggest that measures for sustainability should also include a broad set of determinants, including the presence of community- or organizational-level partnerships, continued attention to the health being addressed, and replication in other sites [75]. Sustainability has been operationalized according to: 1) maintenance of a program’s initial health benefits, 2) institutionalization of the program in a setting or community, 3) capacity building in the recipient setting or community, and 4) sustainability capacity [76].

#### Example: Community health workers as a sustainable implementation strategy

*Context*: Community health workers (CHWs) are public health workers who work either for pay or as volunteers in a local health care system. CHWs often share language, culture, and lived experiences with their clients. Sustainment of CHW programs usually requires some combination of resources, integration within healthcare systems, and support from supervisors, peers, and providers [77]. In Brazil, the Family Health Strategy relies on CHWs to provide basic primary care to families in their homes. The program has been a core element in Brazil’s primary care system since 1994. The CHWs are part of a core, multidisciplinary team of physicians and nurses [78]. They also have access to other specialists (e.g., psychologists, physiotherapists). The program includes 265,000 CHWs and covers 67% of the population [78]. Over 20 years of research, initially conducted in Ceara, have demonstrated impacts that give health systems confidence in continuing expansion to scale [79, 80].

*Impact*: The Family Health Strategy and its use of CHWs have shifted care in Brazil from more expensive hospital-based care to less costly and effective preventive care. The program has been associated with such impacts as increased rates of immunization, reduced healthcare inequities, increased breastfeeding rates, and fewer avoidable hospitalizations [78].

*Key lessons*: From the Brazilian experience, effective care can be delivered and sustained when CHWs are integrated into a broader multidisciplinary health team (including physicians and nurses). When CHWs share cultural and linguistic backgrounds with the communities they serve, trust, accessibility, and the effectiveness of care delivery can continue.

### De-implementation

Another impact is de-implementation, or stopping practices that are ineffective or harmful, not the most effective or efficient (including cost-effective), or are no longer necessary [81]. Nascent evidence indicates that, like implementation efforts, de-implementation requires active approaches and local champions for success. Intervention de-implementation is a process within a complex system of organizations and individual actors, with bi-directional influences between the intervention and the context from which it is being removed [63]. Extant theories, models, and frameworks (hereafter, “frameworks) help to conceptualize de-implementation [82]. A related concept, mis-implementation, involves the continuation of ineffective interventions (the need for de-implementation) and the premature ending of EBPPs [83]. Among evidence-based programs, almost 40% within US state health departments are discontinued when they should continue [83], i.e., mis-implemented.

#### Example: Lead in gasoline

*Context*: Millions of tons of lead was added to gasoline worldwide since the 1920s to improve vehicle efficiency. The health effects of low-level exposure are increasingly clear and well-documented [84], including impaired neurodevelopment (e.g., lower IQ, decreased attention span), cardiovascular risks, kidney disease, and premature death [85]. In the 1970s, policies and regulations were introduced in many countries to eliminate lead from gasoline. The phase-out was completed in 2021. Following the classic Diffusion Theory [86], there were early (e.g., Japan), middle (e.g., Russia), and late adopters (e.g., Algeria) in the lead elimination process.

*Impact*: Although it took decades, removing lead from gasoline has significantly reduced lead exposure. Eliminating lead in gasoline has increased children’s intelligence, saved lives, and created economic benefits (e.g., health care costs, productivity) [85]. Lead elimination worldwide is complete, but it was slower and more sporadic than necessary, as is often the case with policy de-implementation (also known as policy termination).

*Key lessons*: Even in light of clear scientific evidence of the health risks of lead exposure, de-implementation may lag due to political, economic, and industry resistance. Several properties from Diffusion of Innovation [86] were illustrated in this case study: 1) lead phase out occurred in phases with early, middle and late adopters, 2) adoption was influenced by the relative advantage of the benefits of new policy standards over existing standards, and 3) change is driven in part by economic benefits through reduced health costs and improved productivity.

### Equity

Equity in public health and clinical settings means that all segments of the population, especially the most marginalized, have a fair and just opportunity to be healthy [56]. Inequities can lead to disparities in health outcomes and undermine the overall effectiveness of EBPPs. Public Health 3.0 emphasizes engagement and actions directly affecting the social, environmental, and economic conditions driving health inequities [87]. This comprehensive approach considers the impact of where people live, learn, work, and play, involving intersecting systems such as housing, safety, physical environments, education, and economic stability [88]. Recently, the widespread recognition of racism as a public health crisis and a focus for implementation science has further mobilized action [89–91]. Equity should be a centerpiece for implementation science impacts [64], with participatory approaches yielding tangible, equity-related benefits (e.g., more effective study execution) [92].

#### Example: Participatory budgeting

*Context*: Participatory budgeting is a promising method for involving community partners in the distribution of public funds more equitably. In participatory budgeting, community members directly contribute to the allocation of public funds. The process originated in Brazil in 1989 and is increasingly used across multiple governmental sectors in the United States and globally [93]. The Tacoma-Pierce County Health Department in Washington state (USA) uses a participatory budgeting process [94]. In Tacoma-Pierce County, this process was implemented to support the health department’s focus on equity, which seeks to improve health outcomes in neighborhoods with reduced life expectancy.

*Impact*: In Brazil, participatory budgeting is associated with multiple endpoints, including better access to public services, a reduction in extreme poverty, and decreases in child and infant mortality [95]. Participatory budgeting increases transparency in funding decisions, provides accountability for decisions made by public health agencies, and contributes to more responsive governance and more significant community impact overall.

*Key lessons*: Empowering the community to change funding priorities can lead to increased confidence in the democratic process among community members and greater legitimacy and trust between the community and public health officials. Participatory budgeting can surface overlooked health needs, build capacity to address these needs, and, when implemented effectively, promote sustained commitment to stakeholder engagement. This is a version of highly impactful participatory implementation science [96].

## Enhancing impact: a path forward

Research with impacts beyond the academic requires trust in research and the scholarly process [97]. Recent data show that researchers are trusted more than most other professions. In a 2022 survey in 28 countries, scientists were the 2nd most trusted profession, with 57% of adults surveyed trusting researchers [98]. Yet, trust in scientists varies significantly worldwide. A 2020 survey found that trust in scientists was highest in Australia and New Zealand (62% of respondents expressed high trust) and lowest in Africa (19% expressed high trust) [99]. In some regions, trust has dropped significantly over time. For example, while nearly 80% of Americans support government investments in scientific research [100], trust in scientists declined by 12% from January 2019 through October 2022 [101], and scholars warn of a “deadly rise of anti-science” [102]. Given this decline, the demand for greater research accountability, and pressures on research funding in many countries, researchers must maximize and document the impacts of implementation science beyond academia.

Frameworks provide a systematic roadmap for guiding implementation science study design, measures, data collection, data analysis, and outputs (e.g., Reach, Effectiveness, Adoption, Implementation, and Maintenance [RE-AIM], Consolidated Framework for Implementation Research [CFIR]) [103]. While over 100 frameworks are used in implementation science [104], few explicitly focus on impacts beyond those typical in a research study (e.g., health outcomes). A notable exception is the framework proposed by Proctor and colleagues (2009), which hypothesizes and proposes demonstrating that successful implementation has a downstream impact on service system outcomes and clinical/population health [105].

Several frameworks and models identify research impacts beyond typical academic metrics [28–32, 106]. For example, the Translational Science Benefits Model provides a framework and benchmarks to measure the impact of scientific discoveries beyond traditional metrics, including 1) clinical and medical benefits; 2) community and public health benefits; 3) economic benefits; and 4) policy and legislative benefits [106]. Most impact frameworks are based on a logic model, commonly used in evaluation to depict how programs or research activities link with shorter-term outputs and longer-term outcomes [107]. Frameworks seek to enhance some combination of accountability, transparency, advocacy, return on investment, and translation speed [108].

We suggest three interconnected pathways to greater impact of implementation science (i.e., funders, other organizations, and individual researchers). The first set of activities involves funders making impact a more explicit requirement in funding announcements on implementation science, knowledge mobilization, and knowledge translation [3, 6, 109]. While it has grown slightly in recent years, we underinvest in implementation science. For example, in the D&I research category, the US NIH invested about 1.75% of its total budget in fiscal year 2023 [110]. This US funding for D&I research is likely to decrease in the coming years due to significant reductions in the NIH budget (the largest public funder of research on the globe) [111]. Knowledge mobilization recommendations have shifted funding priorities toward more D&I science in the UK and other European countries. The second area is for research organizations, particularly universities, to build a “culture of impact” that makes central the use of impact frameworks, including a greater focus on societal impact in hiring and promotion, and better design studies for impact in early stages of the research process [112, 113]. The third area involves individual researchers who can more effectively engage with partners, design for dissemination, and actively disseminate research findings. Wolfenden and colleagues have reported that health policy or practice impact is increased when intervention trialists undertake knowledge translation strategies (e.g., involve end-users, adapt knowledge to the local context, tailor interventions) [114].

Given these challenges and opportunities, *What will elevate the “so what” of implementation science?* Causal pathways between the strategies to promote uptake of an EBPP and a range of impacts (health and otherwise) are complex. This complexity makes it difficult, if not impossible, to attribute a given impact solely to implementation science. However, through our four implementation domains (speed of research translation, scale-up and sustainment, de-implementation, and equity) and the associated examples, it is clear that implementation science methods contribute to a set of impacts of high societal relevance.

We need to actively disseminate research and fundamentally change how knowledge is disseminated and utilized. Greenhalgh distinguished between “letting it happen” and “making it happen” in translating research into practice and policy [115]. Too often, research dissemination has been relegated to the “letting it happen” category, despite a substantial body of literature demonstrating the ineffectiveness of passive dissemination [116, 117]. To improve the dissemination of evidence in practice settings, the push–pull–capacity model posits that for science to affect practice, there must be a combination of the push (a basis in science and technology), the pull (a market demand from practitioners or policymakers) [118], and the capacity (the delivery ability of public health and health care systems) [119] The pull-pull model helps highlight some capacity-related factors that enhance the translation of research to practice (e.g., multipronged approaches, dedicated staff for dissemination) [120–122].

While not exhaustive, we provide ideas to accelerate the impact of research for implementation scientists (Table 4). These recommendations draw upon the broad literature in implementation science (including diffusion theory [86]), lessons from the domains and examples in Table 3, and the combined experience of the authors in implementation research and practice (within clinical care, public health practice, and social work practice). We acknowledge that any actions to enhance research impact are highly contextual—what works will differ across settings or regions of the world. When considering our ideas, the roles of incremental and transformational change are worth noting [123, 124]. Impact based on incremental change primarily involves minor and gradual adjustments to existing practices, but doing these practices more efficiently and effectively. Transformational change is a significant overhaul of an organization, with shifts to systems, culture, and structures (e.g., a substantial change in mission).

**Table 4:** Recommendations to enhance the impact of implementation science, with more transformational changes in bold

*Implementation science principle*	*Recommendation*	*Segments/actors*^*a*^
*Co-production and stakeholder engagement*		
	• Develop and implement a partnership model that focuses on equity and places much of the power with end-users and implementation partners rather than with universities • Partner with diverse individuals and groups to ensure the research is valuable and timely, beginning with the research question(s) being answered • Engage with end users of implementation science throughout the research process • Build collaborations across fields, particularly those outside of the health sector that may have impacts on equity (e.g., economic development, housing, transportation, the arts) • Work closely with policymakers to ensure research findings inform policy decisions	• Researchers • State and local practitioners • Policymakers • Advocates • Funders • Researchers • State and local practitioners • Health system leaders
*Tailored dissemination and knowledge translation*		
	• Establish dedicated entities to rapidly respond to communication priorities, including countering disinformation • Conduct active, multilevel dissemination of research findings • Learn the media landscape (cultivate local, state or national contacts; be available instantly; deliver pithy summaries of research) • When disseminating evidence, account for the potential for implicit biases, negative attitudes towards groups who have been historically marginalized, and structural/historical barriers among marginalized groups • For more extensive research projects, develop robust and comprehensive dissemination plans • Follow principles of effective dissemination that take into account audience preferences, message framing, and appropriate channels (including relevant information in Table 2) • Be aware of, and proactively develop strategies for, the impact of social media for rapidly spreading information (or disinformation) (e.g., COVID-19 vaccination) • Share data with partners in formats that support community-level use and decision-making • Products should reflect the images, stories, and outcomes of interest to populations experiencing inequities	• Researchers • State and local practitioners • Advocates
*Organizational readiness and supportive infrastructure*	• Overhaul academic incentive structures (e.g., promotion and tenure criteria) to reward and place high value on active dissemination, partner engagement, and impactful research • When working with research partners, avoid showing up with pre-determined research plans • Enhance funding for implementation science, in part by showing the value among policymakers • In funding announcements require rigorous dissemination plans, targeting one or more non-research audience • In academic institutions, make dissemination to non-research audiences expected and build units that are dedicated to active dissemination (beyond developing press releases)	• Academic institutions • Funders • Researchers
*Implementation capacity building*		
	• Radically shift the academic training model to focus highly on on-the-job training for workers in health care, public health, and service delivery • Hire and support diverse teams with skills in dissemination and implementation science • Hire and support faculty and staff whose work promises impact, particularly in marginalized communities • Develop and scale-up ongoing training for researchers and partners to improve dissemination skills • In ongoing training programs (e.g., graduate training in public health), conduct bi-directional learning with community partners to illustrate real-world impact	• Academic institutions • Researchers
*Use of implementation impact metrics*		
	• In academic institutions, adopt an impact framework to develop metrics to track research uptake and sustainment outside of academe; realign the organization based on metrics • Evaluate the process of de-implementation • Evaluate the impact of research a dedicated function (with resources) of scientific organizations • Use systems such as Altmetrics to begin to bridge academic products (publications) with societal uptake (media, social media, policy)	• Researchers • Evaluators

^a^Individuals, groups, and partners who are most likely to take action to address the recommendation

The increased impact of research begins with how we *engage* with partners, stakeholders, and end users of evidence. The inequitable power distribution between researchers, clinical partners, public health and service agencies, and the communities served is a significant issue [125]. “Expert” voices of professionals are often emphasized over the lived experiences of community members [125]. Lessons from community-engaged research show that a participatory process enhances the odds of impact with a commitment to mutual goal-setting, implementation of strategies, and continuous improvement [126]. Stakeholder engagement poses challenges, namely the time-intensiveness of the process, potential misalignment between partners’ and academics’ goals, and the privileging of researchers’ voices over those of partners [127, 128]. There is also a need to shift greater influence in implementation science to practitioners and researchers in low- and middle-income countries [129]. In a second category, *dissemination* practices matter. The dissemination of research evidence to non-scientists is enhanced when messages are framed in ways that evoke emotion and interest, and demonstrate usefulness [130]. Organizational readiness is needed to create environments that address the motivations and needs of individuals and organizations seeking to enhance impact. These actions can come in the form of incentive structures (e.g., promotion and tenure guidelines) and organizational capacity (e.g., organizations with expectations for dissemination to non-research audiences are more effective at dissemination [131]). In research institutions, how we *build implementation capacity* among faculty and staff influences our ability to translate science better to practice and policy. And finally, “what gets measured, gets done”—we need to expand *metrics and evaluation* approaches to map and improve implementation science uptake outside research settings. Metrics need to include easier-to-measure indicators (changes in clinical practice) and those more difficult to quantify (changes in social determinants of health).

As we apply these and other ideas more systematically, we must sharpen the focus on equitable impacts for all populations [45], including narratives and metrics around benefits and unintended consequences [132]. As noted in the participatory budgeting example, democratizing health-related research decision-making can provide a greater community voice. To address health equity in implementation science more fully, research organizations and partner agencies should make health equity a core value, develop better tracking systems, build skills among staff, and develop new partnerships [133].

We highlighted that policy and practice environments are complex, and outcomes are the product of various actors, each motivated by different objectives, as highlighted in Table 2. The chain of events leading from a particular research study to a change in policy or practice may be unexpected, with pathways that are difficult to discern. It is often impossible to draw a direct relationship between any specific research finding and a change in policy or practice, mainly because policymaking is an open system where multi-causality is the norm. One of the challenges for our increasing orientation toward impact is mapping these pathways. This mapping can be enhanced by systems science methods that aid in studying complex systems, with heterogeneous and interacting forces [134].

While our essay focuses mainly on the promise and impacts of implementation science, it is essential to remember that academia is about much more than research, and the research function intersects with other parts of a university’s mission. Universities can be engines of innovation and cultural enrichment [1], with significant foci on education and teaching, outreach, economic development, and dissemination of knowledge. Outreach and dissemination are part of the fabric of US land-grant universities within the agriculture extension service. Diffusion theory, the earliest theory in implementation science, originated in research on the diffusion of farming practices, including the use of agricultural extension agents (“change agents” in diffusion theory) [135].

Any discussion of research translation warrants caution, namely that not all research can or should have immediate impacts [136]. Several reasons underly this assertion: 1) research is iterative and often takes time to build an evidence base ready for action, 2) research is sometimes methods-oriented and provides an architecture for the research process but not information that is actionable outside of academe, 3) single studies are seldom definitive—significant impacts often come from aggregated research, and 4) documenting the impact of research translation is challenging, making it difficult, if not impossible, to show causal attribution (rather than contribution to a change). While there is guidance on when evidence is sufficient for D&I (e.g., the quality and quantity of evidence, priority among stakeholders, and availability of resources) [43], these decisions are judgments, not formulaic ones.

## Conclusion

We are at an opportune time to enhance the impact of implementation science on public health, clinical practice, and society. Designing implementation science studies for impact can amplify the goal of achieving greater benefits. This requires new skill sets and use of collaborative approaches that include individual researchers, their organizations, funders, and the communities they aim to benefit. To ensure implementation science is more impactful in filling societal needs, systemic changes are required in academic institutions that often evolve slowly. Navigating the hurdles and the hope of translating research into practice and policy can amplify societal impact, making implementation science more applicable, accessible, and equitable to all.

## Data Availability

Not applicable.

## Abbreviations

ART: Antiretroviral therapy
CHW: Community health worker
D&I: Dissemination and implementation
EBPP: Evidence-based programs and policies
HPV: Human Papilloma Virus
NIH: National Institutes of Health
PEPFAR: President’s Emergency Plan for AIDS Relief
WUNDIR: The Washington University Network for Dissemination and Implementation Research
